# Sleep disturbances correlate with behavioral problems among individuals with Wiedemann-Steiner syndrome

**DOI:** 10.3389/fgene.2022.950082

**Published:** 2022-10-13

**Authors:** Rowena Ng, Hans Tomas Bjornsson, Jill A. Fahrner, Jacqueline Harris

**Affiliations:** ^1^ Kennedy Krieger Institute, Baltimore, MD, United States; ^2^ Department of Psychiatry and Behavioral Sciences, Johns Hopkins University School of Medicine, Baltimore, MD, United States; ^3^ Department of Genetic Medicine, Johns Hopkins University School of Medicine, Baltimore, MD, United States; ^4^ Department of Pediatrics, Johns Hopkins University School of Medicine, Baltimore, MD, United States; ^5^ Faculty of Medicine, University of Iceland, Reykjavik, Iceland; ^6^ Landspitali University Hospital, Reykjavik, Iceland; ^7^ Department of Neurology, Johns Hopkins University School of Medicine, Baltimore, MD, United States

**Keywords:** genetics/genetic disorders, sleep, externalizing behaviors, internalizing behaviors, KMT2A, epigenetics, Wiedemann Steiner syndrome

## Abstract

Wiedemann-Steiner syndrome (WSS) is a rare genetic disorder caused by mutation in *KMT2A* and characterized by neurodevelopmental delay. This study is the first prospective investigation to examine the sleep and behavioral phenotypes among those with WSS through parent-informant screening inventories. A total of 24 parents of children/adults with WSS (11F, Mean age = 12.71 years, SD = 8.17) completed the Strengths and Difficulties Questionnaire (SDQ) and 22 of these caregivers also completed the Modified Simonds and Parraga Sleep Questionnaire (MSPSQ). On average, the majority of those with WSS (83%) were rated to show borderline to clinical level of behavioral difficulties on the SDQ. Approximately 83% were rated in these ranges for hyperactivity, 63% for emotional problems, and 50% for conduct problems. When applying prior published clinical cut-off for risk of sleep disturbance among those with neurodevelopmental disorders, over 80% of our sample exceeded this limit on the MSPSQ. Largely, caregivers’ ratings suggested restless sleep, rigid bedtime rituals, sleep reluctance and breathing through the mouth in sleep were most consistent problems observed. Partial correlations between sleep and behavioral domains showed elevated emotional problems were associated with parasomnia characteristics after controlling for age. Daytime drowsiness and activity were associated with more hyperactivity. Those with more night waking problems and delayed sleep onset were rated to show more severe conduct problems. Overall, these findings suggest dysfunctional sleep behaviors, hyperactivity, and affective problems are part of the neurobehavioral phenotype of WSS. Routine clinical care for those affected by WSS should include close monitoring of sleep and overactive behaviors.

## Introduction

Wiedemann-Steiner syndrome (WSS) is a rare Mendelian disorder of epigenetic machinery (MDEM), a group of genetic conditions defined by disrupted components of the epigenetic regulatory system (e.g., writing, erasing, and reading histone or chromatin marks, and chromatin remodeling) (Fahrner and Bjornsson, 2014; Fahrner and Bjornsson, 2019). WSS is caused by mutations in *KMT2A* (Jones et al., 2012), a writer which catalyzes histone methylation at H3 lysine K4 (H3K4). Cardinal features of WSS include hypertrichosis, distinctive facial features, and growth retardation (Jones et al., 2012; Miyake et al., 2016; Aggarwal et al., 2017). Intellectual disability or developmental delay is nearly universal among those with WSS (Baer et al., 2018; Sheppard et al., 2021).

Dysregulated histone methylation including H3K4 has been hypothesized as a possible pathophysiological mechanism for select developmental psychopathology, specifically psychotic disorders (Shen et al., 2014; Vallianatos and Iwase, 2015; Nesbit et al., 2021) and aggressive behaviors (Vallianatos et al., 2020). Indeed, mice models with deficiency in *KMT2A*- the gene implicated in WSS—show increased aggressive behaviors in addition to reduced dendritic spine density in the ventral hippocampus (Vallianatos et al., 2020). However, to date, the neurobehavioral phenotype associated with individuals with WSS or other MDEMs affected by the family of KMT genes, which catalyzes H3K4 methylation, is not well understood given the rarity of these syndromes.

Specifically, the limited literature involving those with WSS largely comprise of case reports or clinical case series, typically reliant on a retrospective review of medical records. As such, there has been mixed findings regarding the prevalence of problem behaviors, affective symptoms and clinical features. In a recent observational study of 104 individuals with WSS (Sheppard et al., 2021), ranging from 4 months to 43 years of age, about 46% of their sample were reported to show poor sleep, 44% with hyperactivity and 33% with aggressive behaviors. These observed rates diverged from those reported in a meta-analysis (Chan et al., 2019) with 127 individuals with WSS with a little over 10% with sleep problems, about 6% with hyperactivity, and approximately 15% with aggression. However, discrepancies in reported findings are likely due to methodological issues, including the lack of consistency in assessing these areas or documenting evaluation outcomes from an individual patient-level leading to missing phenotypic information. As such, it is unclear from medical records whether these behaviors were not assessed versus not clinically present. To better characterize the neurobehavioral phenotype associated with WSS, prospective research will need to utilize a consistent battery of reliable screening tools that has been validated and psychometrically supported among those with intellectual disability and neurodevelopmental disorders. Notably, given recent literature demonstrating potential feasibility of postnatal rescue of functional outcomes in MDEMs with overlapping molecular mechanisms and phenotypes to WSS—such as Kabuki syndrome (Bjornsson et al., 2014; Benjamin et al., 2017)—comprehensive phenotyping efforts will be essential to determine targets for epigenetic therapies, in addition to informing clinical care and development of syndrome-specific behavioral interventions.

The goal for this study is to better define the sleep and behavioral profiles associated with individuals with WSS by utilizing screening measures that have been previously published among those with neurodevelopmental disorders, including autism spectrum disorder, developmental delays, and intellectual disability (Modified Simonds and Parraga Sleep Questionnaire, MSPSQ; Strengths and Difficulties Questionnaire, SDQ). From direct clinical interactions with patients, we anticipated elevated sleep difficulties although we had no specific predictions whether unique patterns would emerge in distinct sleep characteristics (e.g., sleep breathing disorder, parasomnia, sleep onset, waking, sleep anxiety). Given recent case series involving those with WSS suggest potentially elevated patterns of emotion and behavior regulation difficulties (Ng et al., 2022), we hypothesized a relatively high rate of at-risk or clinically significant problem behaviors in our sample. Additionally, previous studies have suggested that sleep dysfunction correlates with increased rates of maladaptive behaviors in both typically developing children and those with neurodevelopmental disabilities (Stein et al., 2001; Esbensen et al., 2018) as such, we expected this correlation would also be present in the WSS sample.

## Materials and methods

### Participants

A total of 24 parents of individuals with molecularly-confirmed WSS (11F, M_age_ = 12.71 years, SD = 8.17, range = 4–33) completed a series of surveys on their sleep functioning and behavioral functioning. All participants identified their ethnic background as White. Table 1 outlines sociodemographic and genetic test information of our sample. Families of those with WSS were recruited through international patient advocacy groups. The final sample in this study included caregivers that reported English proficiency and provided a copy of their child’s genetic test results. All genetic records were reviewed to confirm the WSS diagnosis. All parents reported residency in the United States with the exception of three from Australia, Netherlands, and Canada. The majority of our sample completed whole exome sequencing (*N* = 19). Nineteen participants had *de novo* variants, two inherited from parental mosaicism, and three were of unknown inheritance. All but two participants had pathogenic or likely pathogenic variants. Exclusionary criteria included detection of additional genetic syndromes known to impact neurodevelopment. This study was approved by the Institutional Review Board at Johns Hopkins University and in accordance with the Helsinski Declaration. Informed consent and/or assent were obtained by patient’s legal guardian and/or the patient prior to inclusion in the study.

**TABLE 1:** Participant characteristics.

	WSS sample
*N*	24
Mean age in years (sd)[range]	12.71 (8.17) [4.21–33.93]
Sex	11 females
Country of origin	
United States	21
Australia	1
Canada	1
Netherlands	1
Genetic testing	
Inheritance	
*de novo*	19
mosaic	2
unknown	3
Pathogenicity	
pathogenic	21
likely pathogenic	1
variant of uncertain significance	2
Test type	
whole exome sequencing	19
single gene panel	3
cornelia de lange	1
research panel	1

### Procedure and materials

All parent respondents completed a research intake questionnaire that included questions regarding their child’s history of participating in behavioral intervention for problem behaviors or affective concerns, and self-injurious behaviors (SIB). Parents provided yes/no responses to indicate endorsement of SIB and treatment engagement.

#### Strengths and Difficulties Questionnaire

Caregivers also completed the SDQ (Goodman, 1997), which is a commonly used inventory used across medical and mental health clinics to screen for problem behaviors that are applicable for individuals age 2 years and older. Respondents are instructed to rate their child’s behaviors on a three-point Likert scale (0 = Not true, 1 = Somewhat true, 2 = Certainly true). The inventory includes 25 rating items, which yields five scales (Emotional Problems, Conduct Problems, Hyperactivity, Peer Problems, and Prosocial Behaviors) in addition to a total composite. Elevated scores on all scales, with the exception of Prosocial Behaviors, reflect more problems within the domain. In contrast, Prosocial Behaviors is a strength-based scale, such that higher scores trend towards with normal limits. The impact supplement includes items rated on four-point Likert scale (0 = Not at all, 1 = Only a little, 2 = Medium amount, 3 = A great deal) that focuses on the extent the behaviors cause distress to the child and functional impairment across settings (home life, friendships, classroom learning or work, and leisure activities). We applied the original three-band categorization, which allows classification of scores (Within normal limits, Borderline, Clinically Significant) by aggregating total raw scores per scale and applying the band cut-offs.

Externalizing and Internalizing scales were also computed. The Externalizing scale constitutes the sum of Conduct Problems and Hyperactivity scales, and the Internalizing scale is comprised of the Emotional Problems and Peer Problems scales. Externalizing and Internalizing scales are interpreted as a continuous dimensional measure to assess risk for psychopathology and are not categorized by clinical cut-off ranges (Goodman and Goodman, 2009). Of note, clinical use of the parent-informant SDQ in screening for mental health concerns has been supported among children with intellectual disability (Kaptein et al., 2008). Validity of the borderline clinical cut-off for the SDQ among those with intellectual disability has also been documented (Rice et al., 2018).

#### Modified Simonds and Parraga Sleep Questionnaire

Of our total sample of 24, 22 caregivers completed the MSPSQ (Simonds and Parraga, 1982), which has been used in screening sleep disturbances for individuals 5 years and older. This measure has been used among caregivers of in individuals with neurodevelopmental disorders including intellectual disability (Wiggs and Stores, 1996; Wiggs and Stores, 2004) and autism spectrum disorder (Johnson et al., 2012) as well as other MDEMs like Kabuki syndrome and KAT6A syndrome (Smith and Harris, 2021; Rapp et al., 2022). The MSPSQ includes 51 items that require caregivers to provide a mix of qualitative responses to open-ended questions, and ratings on a Likert scale or forced choice format (yes/no). Specifically, of these, 36 items emphasized on frequency of a sleep behavior (1 = Never, 2 = About once a month, 3 = A few times a month, 4 = Once or twice a week, 5 = Many times a week or daily) or duration to fall asleep or return to sleep (1 = Few minutes, 2 = Up to 30 min, 3 = Up to 60 min, 4 = 1–2 h, 5 = Over 2 h). The other 15 items pertained to sleep environment (e.g., co-sleeping), history of seeking medical advice, perception of sleep problem, and sleep quality. Internal consistency of the MSPSQ among individuals with intellectual disability is strong (Cronbach’s α = 0.80, Maas et al., 2011) and adequate among those with autism spectrum disorder (Cronbach’s *α* = 0.67, Johnson et al., 2012). Cross inventory investigations have found strong correlations between MSPSQ with other common inventories used in the assessment and treatment of children with neurodevelopmental disorders and sleep disturbances including the Children’s Sleep Habits Questionnaire (Johnson et al., 2012) and Sleep Disturbance Scale for Children (Maas et al., 2011). This study focused on the same frequency rating items and sleep factors (Parasomnia, Sleep Disordered Breathing, Sleep Anxiety, Bedtime Resistance, Night Waking) published in Johnson et al. (2012), with the exception of the Daytime Sleepiness factor given Cronbach alpha was low (0.19). The two items that comprised this scale were reviewed individually for qualitative purposes rather jointly as a single factor (Seems drowsy during the day but can stop himself/herself from sleeping; During the day, appears more active than other children).

### Data strategy

Frequency analysis was used to determine the proportion of participants with a history of behavior intervention engagement and self-injurious behaviors as indicated on the research intake questionnaire. Similarly, we examined the percentage of our sample who sought medical advice, reported a diagnosis of sleep apnea, received treatment for poor sleep, and identified their child’s sleep as problematic based on force choice (yes/no) and qualitative responses on the MSPSQ. Subsequently, descriptive analyses were conducted to determine mean ratings across sleep factors on the MSPSQ and across subscales on the SDQ. Additionally, the proportion of participants falling within borderline to clinically significant band classifications across the SDQ scales were computed to determine relative risk for behavioral or psychosocial dysfunction in our sample. Likewise, proportion of poor sleepers was estimated by adopting the MSPSQ total score cut-off of 56, which yielded sensitivity of 0.85 and specificity of 0.70 with an agreement of 89.7% with the Children’s Sleep Habit Questionnaire in a previous study (Johnson et al., 2012). Finally, bivariate correlations were first conducted to examine associations between sleep factors and internalizing/externalizing scales. Given age was correlated with daytime overactivity (*r* = −0.44, *p* = 0.036) and internalizing symptoms (*r* = 0.52, *p* = 0.008), partial correlations were subsequently applied to determine if the pattern of results persist while controlling for participant’s age.

## Results

### Sleep profile of individuals with WSS

Of the 22 respondents who completed the MSPSQ, 14 or 63.63% of patients have sought medical advice or treatment for sleep with five (22.72%) on medication currently, and 12 or 54.54% identify their child as having a current sleep problem. All of the 14 patients who sought medical consultation were reported to have had some form of treatment for sleep concerns. Specifically, nine caregivers reported that their child was diagnosed with a form of sleep apnea (40.90%). Six of those had surgical intervention (27.27%) and two were on mechanical treatment (9.09%).

Table 2 outlines the average MSPSQ scale scores and proportion of participants endorsing sleep symptoms. Collectively, when all items are considered, approximately 86% of our sample were considered to show dysfunctional sleep utilizing the 56 total score threshold (Johnson et al., 2012). The most frequently observed sleep problems were breathing through mouth, waking up during the night, restless sleep, need for bedtime ritual, need for security object, and reluctance going to sleep. Of note, one caregiver did not rate an item regarding feeling afraid when going to sleep.

**TABLE 2:** Distribution of reported sleep difficulties on the MSPSQ among individuals with WSS (*N* = 22).

	Mean (SD)	Proportion of sample endorsing sleep symptoms		
		“Never” to “about once a month”	Few times a month	“Once or twice a week” to “many times a week”
**Bedtime Resistance (5 items, Possible Score Range: 5–25)**	13.55 (5.03)			
How often does your child resist or struggle with you around bedtime	3.23 (1.71)	8 (36.36%)	4 (18.18%)	10 (45.46%)
Doesn’t want to go to bed because s/he is afraid^a^	1.48 (1.12)	18 (85.74%)	1 (4.76%)	2 (9.53%)
Insists on sleeping elsewhere instead of his/her bed^a^	2.36 (1.81)	14 (63.63%)	1 (4.54%)	7 (31.82%)
Insists on bedtime rituals before sleep^a^	3.50 (1.89)	8 (36.36%)	1 (4.54%)	13 (59.09%)
Reluctant to go to bed^a^	3.09 (1.65)	9 (40.90%)	3 (13.63%)	10 (45.46%)
**Sleep Anxiety (5 items, Possible Score Range: 5–25)^a^**	11.27 (4.24)			
Expresses fear that if s/he goes to sleep they might die	1.09 (0.29)	22 (100%)	0 (0%)	0 (0%)
Needs security object before s/he goes to sleep	2.91 (1.90)	11 (50%)	0 (0%)	11 (50%)
**Parasomnia (11 items, Possible Score Range:11–55)**	19.95 (5.95)			
Talks in Sleep	1.64 (1.13)	17 (77.27%)	2 (9.09%)	3 (13.63%)
Walks in Sleep	1.05 (0.21)	22 (100%)	0 (0%)	0 (0%)
Grinds teeth in sleep	2.23 (1.71)	14 (63.63%)	1 (4.54%)	7 (31.82%)
Bangs head at night	1.55 (1.14)	18 (81.81%)	2 (9.09%)	2 (9.09%)
Has quick movements of arms or legs during sleep	1.77 (1.44)	17 (77.27%)	2 (9.09%)	3 (13.63%)
Moves around a lot in bed during sleep	3.23 (1.77)	8 (36.36%)	3 (13.63%)	11 (50%)
Bites tongue during sleep	1.23 (0.86)	21 (95.45%)	0 (0%)	1 (4.54%)
Wets bed during sleep	1.82 (1.59)	17 (77.27%)	1 (4.54%)	4 (18.18%)
Wakes in the night complaining of nightmares or frightening dreams and seems quite anxious	1.55 (1.22)	19 (86.36%)	1 (4.54%)	2 (9.09%)
Wakes during the night screaming in terror	1.41 (0.95)	20 (90.90%)	1 (4.54%)	1 (4.54%)
Sweats a lot during sleep	1.95 (1.49)	16 (72.72%)	2 (9.09%)	4 (18.18%)
**Sleep Disordered Breathing (5 items, Possible Score Range: 5–25)**	11.32 (3.72)			
Snores loudly during sleep	2.50 (1.76)	12 (54.54%)	2 (9.09%)	8 (36.37%)
Seems to repeatedly stop breathing for periods of time lasting up to 30 s during sleep	1.27 (0.93)	20 (90.90%)	1 (4.54%)	1 (4.54%)
Sleeps with head tipped right back	2.27 (1.69)	14 (63.63%)	1 (4.54%)	7 (31.82%)
Breathes through mouth rather than nose when asleep	3.90 (1.44)	3 (13.63%)	5 (22.72%)	14 (63.63%)
Complains of headaches on waking up	1.40 (0.79)	18 (81.81%)	4 (18.18%)	0 (0%)
**Daytime Drowsiness (Possible Score Range: 1–5)**				
Seems drowsy during the day but can stop himself/herself from sleeping	2.14 (1.64)	14 (63.63%)	3 (13.63%)	5 (22.73%)
**Daytime Overactivity (Possible Score Range: 1–5)**				
During the day, appears more active than other children	2.68 (1.81)	12 (54.54%)	2 (9.09%)	8 (36.37%)
**Night Waking 2 items, (Possible Score Range: 2–10)**	4.59 (1.73)			
How often does your child wake up during the night?	3.27 (1.42)	8 (36.36%)	5 (22.72%)	9 (40.90%)
		“Few minutes” to “Up to half an hour”	Up to 1 hour	“Between 1 and 2 h” to “Over 2 h”
How long does it usually take for your child to fall back asleep?	1.32 (0.89)	18 (81.81%)	3 (13.63%)	1 (4.54%)
**Sleep Onset Delay (Possible Score Range: 1–5)**				
How long does it take your child to fall asleep at night?	2.41 (0.95)	14 (63.63%)	4 (18.18%)	4 (18.18%)

a Sleep Anxiety Factor also includes item on anxiety going to bed and bedtime rituals, both of which are included in the Bedtime Resistance Factor. One caregiver did not complete the item on being afraid to go to bed.

### Behavioral functioning among individuals with WSS

Of our entire sample, one caregiver did not respond to the item regarding behavior intervention history. Eleven of the remaining 23 respondents (47.82%) reported that their child has a history of engagement in behavior intervention, and four (17.39%) report a history of ABA therapy. Five participants (20.83%) report concerns with self injurious behaviors.

Table 3 includes average SDQ scale score and proportion of participants falling within the three band classification (Within normal limits, Borderline, Clinically Significant). On average, our sample scored in the clinically significant range in Total Difficulties index, and in the Emotional Problems, Hyperactivity, and Peer Problems scales. Caregivers also rated our sample in the clinical level for functional impact of these problem behaviors. On average, Conduct Problems and Prosocial Behaviors did not meet clinical categorization.

**TABLE 3:** Average ratings on the SDQ and proportion of individuals with WSS with elevated problem behaviors using the three band categorization (*N* = 24).

	Mean (SD)	Borderline three-band categorization	Clinical level three-band categorization	Possible score range
Total difficulties	19.42 (5.23)	14–16	17–40	0–40
Emotional problems	4.67 (3.08)	4	5–10	0–10
Conduct problems	2.54 (1.56)	3	4–10	0–10
Hyperactivity	7.67 (1.90)	6	7–10	0–10
Peer problems	4.54 (1.88)	3	4–10	0–10
Prosocial behaviors	6.67 (1.92)	5	0–4	0–10
Externalizing problems	10.21 (2.75)	---	---	0–20
Internalizing problems	9.21 (4.19)	---	---	0–20
Total impact	4.96 (2.15)	1	2–10	0–10

Note. Elevated ratings across SDQ, scales reflect more problems with the domain with the exception of Prosocial Behaviors, which is a strengths-based scale. Higher ratings on this scale trend towards normal limits. The three-band categorization was utilized to determine if clinical level of symptoms was met (Goodman, 1997). Externalizing and Internalizing Problems scales are interpreted as a continuous dimensional measure to assess risk for psychopathology and are not categorized by clinical cut-off ranges (Goodman and Goodman, 2009).

**FIGURE 1:**
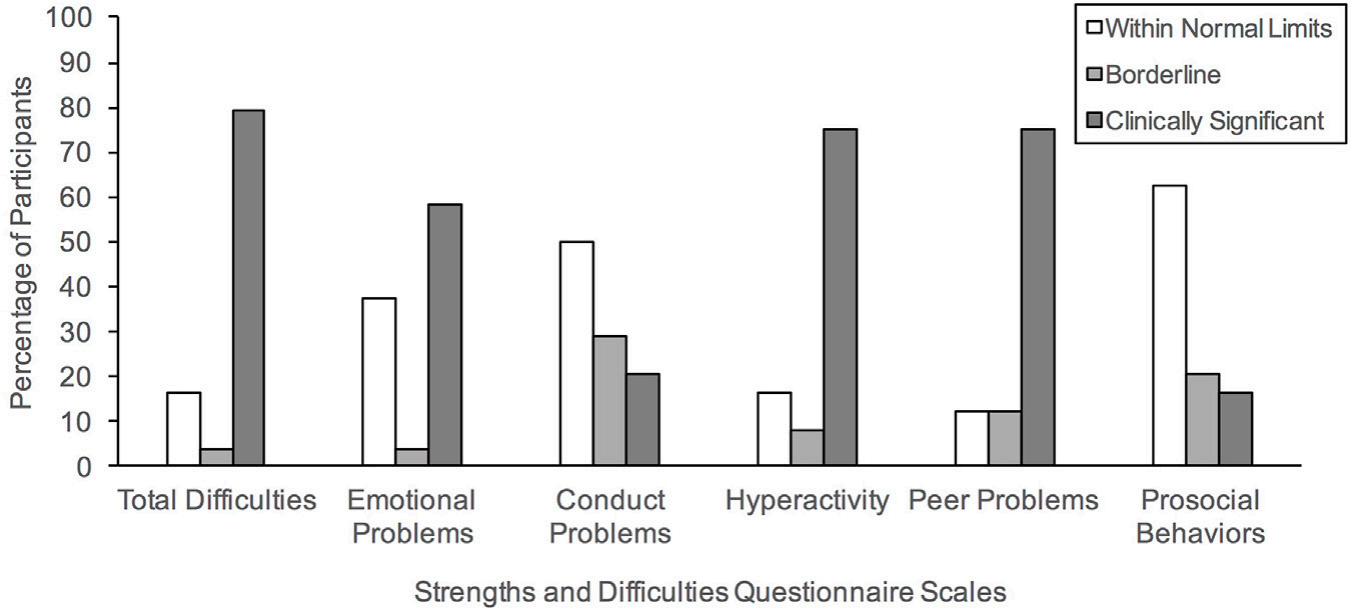
Percentage of our clinical sample meeting the three band categories (Within Normal Limits, Borderline, Clinically Significant) based on the parent-informant version of the Strengths and Difficulties Questionnaire.

As illustrated in Figure 1, approximately 83.33% of our sample were rated to show borderline to clinically significant problematic behaviors, as indicated by the Total Difficulties index (Borderline: 4.16%, Clinically Significant: 79.16%). Specifically, 83.33% fell within these ranges for Hyperactivity (Borderline: 8.33%, Clinically Significant: 75%), 87.50% for Peer Problems (Borderline: 12.50% Clinically Significant: 75%), 62.50% for Emotional Problems (Borderline: 4.16%, Clinically Significant: 58.33%), and 50% for Conduct Problems (Borderline: 29.16%, Clinically Significant: 20.83%). In contrast, 37.50% were rated to show problems with Prosocial Behaviors (Borderline: 20.83% Clinically Significant: 16.66%). The entire sample of caregivers reported borderline to significant functional impairment resulting from these problem behaviors.

### Associations between sleep and behavioral functioning

Tables 4, 5 show the resulting correlations observed between sleep factors, medical consultation, and perception of sleep problems with internalizing and externalizing behaviors.

**TABLE 4:** Bivariate correlations between sleep features and problem behaviors (*N* = 22).

	Bedtime resistance	Sleep anxiety	Parasomnia	Sleep disordered breathing	Daytime drowsiness	Daytime overactivity	Night waking	Sleep onset delay	Medical advice or treatment	Sleep problems
Externalizing behaviors	0.36	0.19	0.29	0.18	**0.43 (0.042)**	**0.50 (0.016)**	0.36	0.40	0.10	0.18
Hyperactivity	0.39	0.20	0.10	0.16	0.26	**0.59 (0.003)**	0.05	0.17	0.009	0.03
Conduct problems	0.16	0.09	0.38	0.11	**0.44 (0.040)**	0.15	**0.57 (0.005)**	**0.48 (0.021)**	0.16	0.28
Internalizing behaviors	−0.02	0.14	0.34	0.09	0.31	−0.14	0.28	0.34	**0.44 (0.038)**	0.27
Emotional problems	−0.03	0.14	0.39	0.12	0.41	0.05	0.29	0.27	**0.51 (0.015)**	0.22
Peer problems	−0.001	0.07	0.05	−0.006	−0.03	−0.41	0.11	0.29	0.08	0.21

Note. Correlations that reached significance (*p* < 0.05) are bolded with the resulting *p*-value in parentheses.

**TABLE 5:** Bivariate correlations between sleep features and problem behaviors while controlling for age (*N* = 22).

	Bedtime resistance	Sleep anxiety	Parasomnia	Sleep disordered breathing	Daytime drowsiness	Daytime overactivity	Night waking	Sleep onset delay	Medical advice or treatment	Sleep problems
Externalizing Behaviors	0.28	0.11	0.27	0.17	**0.51 (0.018)**	**0.43 (0.047)**	**0.48 (0.024)**	**0.47 (0.030)**	0.10	0.25
Hyperactivity	0.21	0.02	0.05	0.16	**0.45 (0.039)**	**0.48 (0.026)**	0.28	0.33	0.008	0.17
Conduct Problems	0.27	0.17	0.41	0.13	0.42	0.26	**0.54 (0.010)**	**0.47 (0.029)**	0.17	0.26
Internalizing Behaviors	0.21	0.36	**0.45 (0.038)**	0.14	0.28	0.08	0.17	0.33	**0.52 (0.015)**	0.22
Emotional Problems	0.14	0.20	**0.47 (0.028)**	0.16	0.39	0.26	0.20	0.24	**0.56 (0.008)**	0.17
Peer Problems	0.19	0.23	0.10	0.02	−0.11	−0.29	0.005	0.26	0.09	0.16

Note. Correlations that reached significance (*p* < 0.05) are bolded with the resulting *p*-value in parentheses.

Overall, caregivers who sought medical advice or treatment for sleep have children who present with more internalizing behaviors, specifically emotional problems. Daytime drowsiness and activity level were associated with increased externalizing behaviors. Parents reported more frequent daytime drowsiness with more conduct problems; and high activity level was also correlated with hyperactivity. Elevated conduct problems were also associated with night waking, and delayed sleep onset.

**FIGURE 2:**
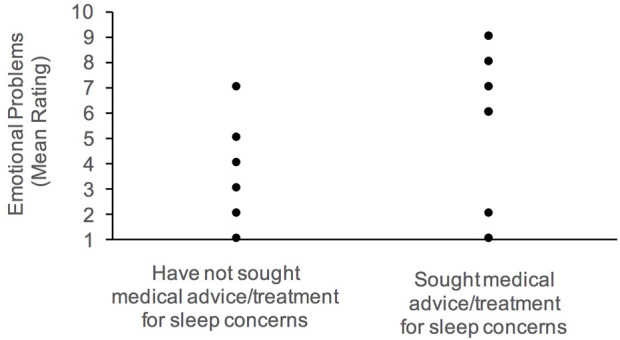
Association between emotional problems and history of medical attention for sleep concerns.

**FIGURE 3:**
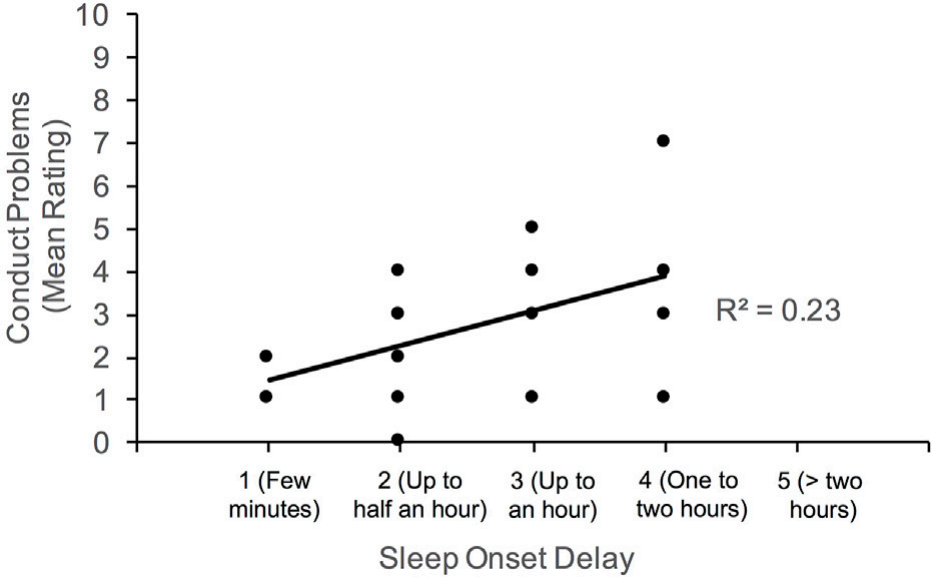
Association between conduct problems and sleep onset delay.

**FIGURE 4:**
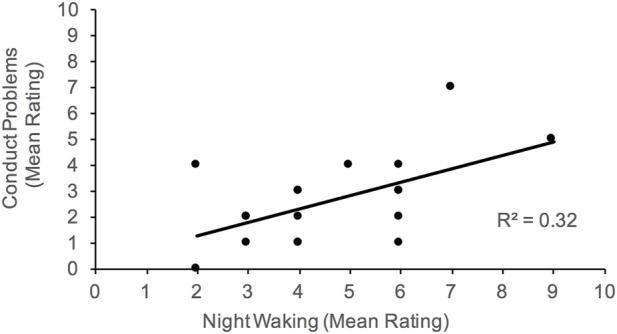
Association between conduct problems and night waking. Higher scores on night waking reflect more problems with sleep maintenance.

Given the large age range of our participants, partial correlations were subsequently computed to determine if the associations noted above persist between sleep and behavioral functioning after controlling for age. Generally, the pattern of results noted above were observed; however, the positive correlation between conduct problems and daytime drowsiness weakened to marginal significance (*p =* 0.055). Figures 2–4 illustrate the associations between sleep and behavioral functioning variables that persist when age was accounted for. Interestingly, after controlling for age, associations between hyperactivity and daytime drowsiness, and between increased parasomnia with emotional problems strengthened. Peer relations, a scale subsumed under Internalizing Behaviors composite, was not linked to any sleep variables.

## Discussion

Our study of individuals with molecularly confirmed WSS show extremely high rates of sleep problems that are largely behavioral in nature, and high levels of problem behaviors. Although sleep apnea was highly represented in our sample, parent ratings across sleep-disordered breathing items reflect low endorsement of symptoms in the last month. This likely reflects sleep apnea that improved following surgical and/or mechanical interventions. Daytime sleepiness/activity, night waking and late sleep onset were associated with externalizing behaviors, while more frequent parasomnias was related to more internalizing features.

Overall, our results suggest sleep dysfunction is common among those with WSS, in line with recent cross-syndrome investigations involving rare genetic disorders including Smith-Magenis, Fragile X, Angelman, Prader-Willi, Cornelia de Lange, CHARGE, Williams, KAT6A, and Kabuki syndromes (Angriman et al., 2015; Kaufmann et al., 2017; Agar et al., 2021; Budimirovic et al., 2021; Smith and Harris, 2021; Veatch et al., 2021; Rapp et al., 2022). Within our sample, sleep apnea was reported at a relatively high rate (40.90%) compared to the recent study by Sheppard et al. (2021) (24.7%). However, it should be noted that parents in our study did not specify the type of sleep apnea diagnosed, whereas, the multisite observational investigation by Sheppard and colleagues (2021) focused on the prevalence of obstructive sleep apnea. As shown in Table 2, the distribution of responses across items reveal low endorsement of current sleep-breathing problems, parasomnias, sleep anxiety, or delayed sleep. Instead, items that are more consistently endorsed largely consisted of bedtime resistance, rigidity in nighttime routines, and restlessness in sleep. In addition, caregivers in our study report frequent breathing through the mouth which may reflect a residual sleep-breathing symptom after corrective interventions. Interestingly, despite over 80% of our sample meeting clinical cut-off for dysregulated sleep on the MSPSQ, a little over half our sample perceived sleep as a problem and only about two-thirds sought medical consultation. Findings highlight the need for clinicians to thoroughly and regularly evaluate for sleep problems—including but not limited to sleep breathing disorders–when working with this population. Provision of professional education to caregivers on sleep disturbances and key signs to monitor should be considered as part of their care management.

Diagnostic assessment for sleep functioning should include detailed review of sleep behaviors, environment and routine in addition to formal polysomnography. While all of the participants in our sample who sought medical consultation took medication and/or had surgical or mechanical interventions, none of the caregivers reported engaging in behavioral therapies for sleep. Given behavioral interventions (e.g., supporting parents in bedtime routines, use of reinforcements, application of objective sleep metrics such as sleep diaries) have shown promising results among adults and children with comorbid neurodevelopmental disorders and sleep difficulties (Thackeray and Richdale, 2002; Van de Wouw et al., 2012; Meltzer and Mindell, 2014; Priday et al., 2017), treatment for individuals with WSS should integrate behavioral strategies to support caregivers. Review of patients’ current medication regimen should also be carefully approached by clinicians to ensure sleep disruptions, or likewise affect/behavior dysregulation, are not potential side effects.

Problem behaviors were extremely common in our sample of individuals with WSS. More than 75% of individuals with WSS had clinically significant levels of total behavioral difficulties, hyperactivity, and problems with peers. More than half had clinically significant issues with emotional problems. These rates are more elevated compared to those previously reported by Baer et al. (2018) (behavioral disorder: 32.2%), Chan et al. (2019) (hyperactivity and ADHD: less than 10%, aggression: little less than 15%, anxiety: less than 10%, emotion and behavior dysregulation: less than 10%, oppositional defiant disorder: less than 5%), and Sheppard et al. (2021) (hyperactivity: 44.3%; aggression: 33%). Discrepancies in findings likely reflect methodological differences as these observational studies and case series largely constitute a retrospective review of medical records, which may not comprehensively document psychosocial difficulties or utilize standardized screening measures to quantify the extent of behavioral challenges. To our knowledge, this is the largest study to date that directly assesses maladaptive behaviors in WSS.

Notably, the number of participants in our sample with a history of behavioral intervention is disproportionately low, relative to the rate of those with problem behaviors. It is possible that psychotropic medication use, which was not inquired in our study, was favored by these families as a primary therapeutic approach for externalizing and affective problems. It is also possible that the impact of these problematic behaviors is under-recognized and individuals are not receiving proper and adequate intervention. Incredibly, 23 of the 24 caregivers surveyed rated the total functional impact of maladaptive behaviors in the clinically significant range with the one remaining falling in the borderline range. This suggests that behavioral issues, especially ADHD behaviors such as hyperactivity, are an incredibly important feature of this disorder and are likely under-recognized and under-treated.

Clinical care for those with WSS should involve regularly evaluating behavioral, anxiety and mood concerns given the deleterious effect on day-to-day functioning across environments, social relationships, and quality of life. Moreover, prospective investigations will benefit from applying a combination of informant report inventories (e.g., parent, teacher, and self-report) to determine the extent mood, anxiety and behavior regulation problems are stable across settings or may be externally driven. Given hyperactive behaviors was most unanimously observed among our study sample, development of clinical trials for WSS may consider this factor as a potential outcome measurement. Developmental and multi-methodological approaches (e.g., integration of neurophysiological, imaging and behavioral measurements) toward investigating behavioral functioning in WSS will be essential given neurogenesis of neural substrates, including prefrontal and limbic regions that are involved with emotion processing and self-regulation skills, may unfold at different trajectories among those with MDEMs. Altogether, comprehensive investigations dedicated to defining the sleep and neurobehavioral phenotypes of those with WSS will be central in building targeted treatment plans specific to this genetic condition and in refining outcome markers for epigenetic therapies.

Sleep disturbances that were largely behavioral in nature were associated with increased scores on the SDQ hyperactivity and conduct problems scales, while parasomnia characteristics were correlated with more emotional problems. The observed associations are not uncommon among those with neurodevelopmental disorders, including intellectual disability (Didden et al., 2002; Rzepecka et al., 2011) and (Rzepecka et al., 2011; Leader et al., 2022); and genetic conditions (Esbensen, 2016; Esbensen and Schwichtenberg, 2016; Zambrelli et al., 2016). As noted above, these results implicate the need to promptly assess and treat sleep and psychosocial concerns, as both may bidirectionally affect the other. It remains unknown whether specific MDEMs may be at greater risk for select sleep disorders (e.g., obstructive sleep apnea, insomnia, behavioral sleep disturbances, etc.) as a function of abnormal muscular activity, craniofacial structures, hypotonia, aberrant circadian rhythm, and/or other congenital malformations; and in turn, poor sleep subsequently leaves these individuals more vulnerable to developmental psychopathology. It is also possible psychosocial maladjustment exacerbates preexisting sleep challenges. Examining both sleep and mental health trajectories jointly can elucidate the pathways in which these clinical features interact over time.

Given the limited existing prospective research involving those with WSS, our study is largely exploratory in nature. As such, less conservative approaches were taken to examine associations between sleep and behavioral functioning, in efforts to offer initial clues to guide subsequent targeted investigations and clinical care. With more conservative data analyses, the risk of missed effects in rare diseases such as WSS may result in missed opportunities for early symptom monitoring and interventions. Future research regarding sleep and/or behavior functioning among individuals with WSS should consider more focused hypotheses and statistical approaches to reduce the risk for Type 1 errors.

Other limitations in our study include heterogeneous sample of individuals with WSS, inclusive of children and adults. Future studies may want to focus on specific developmental periods to determine whether the observed associations between sleep and behaviors vary as a function of maturation and treatments applied. In addition, our study consisted of only those with WSS. Cross-MDEM, patients with sleep disorders without known genetic anomalies, and neurotypical comparison groups should be considered to determine whether observed sleep and mental health associations are unique to WSS relative to other genetic conditions with disrupted epigenetic regulators or compared to those with sleep disturbances broadly. Subsequent investigations with larger and more homogenous developmental samples may consider further exploring sleep and behavioral functioning as a function of gene variants. It should be noted that our study primarily included sleep screening measures based on parent observations. Objective measures of sleep parameters through comprehensive assessments, involving polysomnography, actigraphy, and sleep diaries will be vital in subsequent phenotyping efforts to shed light on the specific symptoms presented by these individuals. Finally, the literature involving the use of SDQ and MSPSQ among children with developmental disabilities remains sparse. More research is necessary to determine the validity of these measures for children with a range of intellectual impairment. Moreover, future investigations with larger samples should consider using more robust inventories to examining the disruptive behaviors that are endorsed by those with WSS, as elevations in Conduct Problems scale may be driven by a few symptoms.

In summary, sleep disturbances, problematic behaviors and affective difficulties are pervasive challenges among those with WSS that warrant close medical attention and thorough evaluations as part of routine clinical care. Sleep behaviors were associated with hyperactivity, conduct problems and affective concerns. Accordingly, when considering treatment plans to address psychopathology among these individuals, clinicians should incorporate behavioral strategies to support sleep hygiene, evaluate the sleep environment, and assess whether select medication regimen may address sleep and problematic behaviors. Finally, from a clinical research standpoint, the universal endorsement of select problem behaviors such as hyperactivity implicate its strong candidacy as a clinical outcome measure. Further systematic investigations dedicated to detailed characterization of sleep and behavioral phenotypes are warranted.

## Data Availability

The data supporting the conclusion of this article are available on request. Due to privacy and ethical concerns, the data are not made available to the public.
